# Sensitive Detection of Kynurenic Acid from Biological Fluids Using a Flexible Electrochemical Platform Based on Gold Nanoparticles and Reduced Graphene Oxide

**DOI:** 10.3390/ijms26030913

**Published:** 2025-01-22

**Authors:** Diana-Gabriela Macovei, Mihaela Tertis, Diana Bogdan, Maria Suciu, Lucian Barbu-Tudoran, Cecilia Cristea

**Affiliations:** 1 Analytical Chemistry Department, Faculty of Pharmacy, “Iuliu Haţieganu” University of Medicine and Pharmacy, 4 Pasteur Street, 400349 Cluj-Napoca, Romania; diana.gabr.macovei@elearn.umfcluj.ro; 2 Isotopic and Molecular Technologies Department, National Institute for Research and Development of Isotopic and Molecular Technologies, 67-103 Donath Street, 400293 Cluj-Napoca, Romania; diana.bogdan@itim-cj.ro; 3 LIME-CETATEA, National Institute for Research and Development of Isotopic and Molecular Technologies, 67-103 Donath Street, 400293 Cluj-Napoca, Romania; maria.suciu@itim-cj.ro (M.S.); lucian.barbu@itim-cj.ro (L.B.-T.)

**Keywords:** kynurenic acid, electrochemical sensor, electrochemical direct detection

## Abstract

Kynurenic acid (KA), a key metabolite of tryptophan (TRP) via the kynurenine pathway, plays a significant role in various physiological and pathological conditions, including neurodegenerative diseases, depression, and schizophrenia. This study aims to develop a flexible and sensitive electrochemical sensor platform for the direct detection of KA in biological fluids. Custom carbon-based electrodes were fabricated using specialized inks and a flexible plastic substrate, followed by functionalization with a composite film of gold nanoparticles, graphene oxide (GO), and polyethyleneimine (PEI). The GO was electrochemically reduced to enhance conductivity and sensitivity for the target analyte. The sensor platform was characterized using cyclic voltammetry (CV), electrochemical impedance spectroscopy (EIS), scanning electron microscopy (SEM), and atomic force microscopy (AFM). An optimized differential pulse voltammetry (DPV) method was employed for KA detection. The developed sensor demonstrated a detection limit of 0.3 nM and was effective across a concentration range of 1 nM to 500 µM. These findings highlight the potential of this electrochemical sensor as a reliable, rapid, and cost-effective tool for KA detection in various biological samples, offering significant advantages over traditional methods in terms of sensitivity and simplicity.

## 1. Introduction

KA is an endogenous compound and a metabolic product of the essential amino acid (TRP) via the kynurenine pathway (KP). Alterations in this pathway are linked to various physiological and pathological conditions, leading to fluctuations in KA levels within biological matrices. Such changes have been correlated with the progression of several diseases, particularly neurodegenerative disorders like Alzheimer’s disease (AD) and Parkinson’s disease (PD) [1]. Notably, KA levels are significantly increased in the cerebrospinal fluid (CSF) of AD patients but reduced in peripheral fluids such as urine and plasma [2]. Conversely, in PD, KA levels are reduced in the CSF [1,3]. These alterations underscore the potential of KA as a biomarker for differential diagnosis and disease progression.

It is important to mention that a positive correlation has been established between two well-known biomarkers already used for differential diagnosis of this type of dementia, namely phosphorylated tau protein (p-tau) and β-amyloid peptide (Aβ), respectively, and kynurenic acid [4,5]. In addition, the metabolite has been reported in the literature as a predictive biomarker for AD [6]. In the second case, the CSF levels of kynurenic acid were reported to be 23% lower in PD subjects [7]. In major depression disorders (MDDs), both peripheral and central levels are altered [8], and one study highlighted the singularity of kynurenic acid as both diagnosis and prediction of treatment response biomarkers out of 20 overlapped biomarkers included in the metabolomic study [9]. A study concluded that, out of many plasma biomarkers evaluated for depressive disorders, kynurenic acid has the biggest diagnostic value, 82.5%. Moreover, promising specificity and sensitivity values were obtained for this biomarker, more exactly 69.9% and 90.0%, respectively [10]. In schizophrenia, the levels of kynurenic acid both in CSF and plasma are increased compared to controls [11].

KA has also been involved in MDDs, where it shows altered levels both centrally and peripherally. It serves as a diagnostic and predictive biomarker, demonstrating significant diagnostic value in depressive disorders. In schizophrenia, KA levels are elevated in both CSF and plasma. The biomarker role of KA is crucial for distinguishing diseases with overlapping etiologies, such as AD and dementia with Lewy bodies (LBD), where KA levels remain unchanged in LBD but vary significantly in AD and PD [12,13,14].

Beyond diagnostics, KA is essential for predicting patient responses to treatment, especially in psychiatry, where therapeutic decisions often rely on empirical models [15]. For instance, in depression, where two-thirds of patients do not achieve symptom remission with first-line antidepressants, robust biomarkers like KA can significantly enhance treatment efficacy [16].

Moreover, KA’s modulation through the KP enzymes is a key research focus for developing new pharmacological treatments. For instance, reducing KA levels is a therapeutic target in schizophrenia, achieved by inhibiting the enzyme kynurenine aminotransferase (KAT) [17]. Conversely, increasing KA levels is beneficial in PD, accomplished by inhibiting kynurenine-3-monooxygenase (KMO). KA also plays a role in renal function, serving as a biomarker for evaluating renal damage and drug excretion. Its properties make it a valuable tool for adjusting medication doses in patients with renal impairments, thereby improving treatment adherence and patient outcomes [18,19].

Recent studies have highlighted KA’s anti-inflammatory and antioxidant effects, its involvement in energy homeostasis, and its role in detoxification processes. These multifaceted roles of KA in physiological and pathological conditions, including neurodegenerative diseases, cancer, and post-Covid syndrome, underline its significance [20,21]. It has been observed that its levels in the human body decrease in some psychiatric disorders but also in cancer [22], while in the case of neurodegenerative diseases and, more recently, in the post-Covid syndrome, increases in concentration have been recorded [23,24,25].

There have been reported traditional methods for KA detection, the most common ones being chromatography methods, such as HPLC [26], UPLC [27], LC [28], and CEC [29]—coupled with one or two types of detectors, for example MS [26,27,29], UV, and FID [28]. A variety of biological fluids have been analyzed through these methods, such as serum, plasma, urine, and CSF. Additionally, there is a commercially available ELISA kit for serum quantification of KA [30]. In terms of immunoassays, an electrochemical immunosensor was also reported in the literature [31]. KA was also quantified by fluorescence spectroscopy [32]. These methods have limitations regarding sensitivity, specificity, and are characterized by the complexity of sample preparation.

In Refs. [26,31,32], KA levels vary depending on the biological fluid, for example, in plasma, KA is secreted in the range of 0.75–11.3 ng mL^−1^ (3.96–59.73 nM); in saliva, the levels are lower, about 0.6 ng mL^−1^ (3.17 nM) [33]. Given the low concentrations of KA in biological fluids, it is crucial to develop detection methods with a high sensitivity. Electrochemical sensors show great potential, as their sensitivity can be enhanced through the use of nanomaterials and composite materials, which expand the electroactive surface area and improve electron transfer capabilities. These advantages underscore the importance of an electrochemical detection platform, such as the one developed in this study. It offers a simpler, faster, and more cost-effective alternative for detecting KA in various biological samples.

The sensor developed in this study provides a novel approach for the direct and sensitive detection of KA in biological samples, achieving a detection limit of 0.3 nM and a broad linear range of 1 nM to 500 µM. Comparison with existing methods [34,35] highlights its competitive performance. Unlike systems incorporating biorecognition elements such as antibodies, which require indirect signal detection, this platform offers direct detection using functionalized carbon electrodes. Functionalization with PEI, AuNPs, and reduced GO enhances conductivity and electrochemical responses, ensuring a high sensitivity.

The flexibility and cost-effectiveness of the custom-printed electrodes add to the sensor’s performance, setting it apart from more complex and expensive platforms like metal–organic frameworks or nanostructured films. While previous studies reported better limits of detection only by using biocomponent-based approaches and indirect detection based on redox probes [31], this sensor achieves comparable sensitivity without relying on complex assembly or indirect testing, making it versatile for practical applications.

Furthermore, the sensor demonstrates excellent performance in real samples, such as serum and saliva, with robustness across various detection scenarios. Another important advantage of the sensor and the method proposed in this study is its validation compared against an optimized HPLC method. Thus, the optimized DPV protocol ensures reliable and reproducible measurements, confirming the sensor’s potential for real-world KA analysis in clinical and research settings.

## 2. Results and Discussions

### 2.1. Development of the Electrochemical Sensor for Direct Electrochemical Detection of Kynurenic Acid

**Scheme 1:**
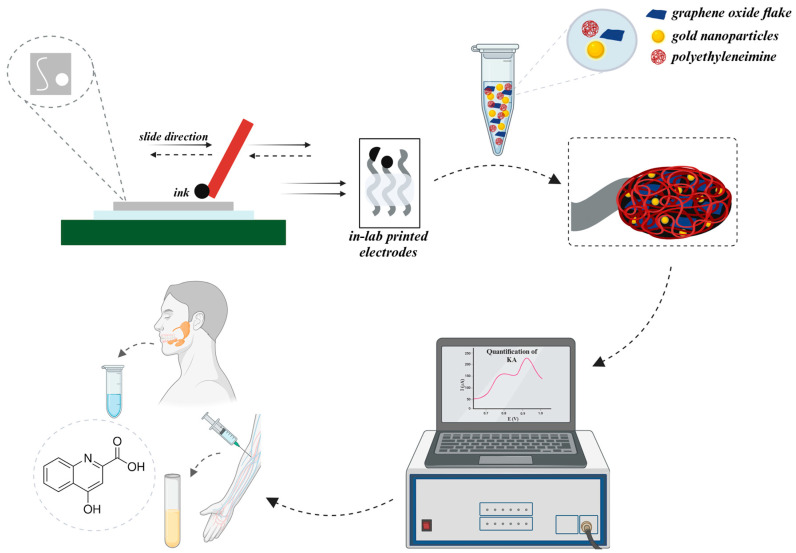
Schematic representation of the main steps in the protocol for developing the electrochemical sensor for KA direct detection (illustration created using the Biorender software (https://www.biorender.com/)).

The development of the electrochemical sensor for direct detection of the biomarker KA in complex biological matrices involved several steps, graphically represented in Scheme 1. Initially, planar electrochemical cells were fabricated in the laboratory, as detailed in Section 3.3. Next, electrode conditioning was necessary to eliminate inter-platform variability using a CV method described in Section 3.4. Only the electrodes that exhibited similar signals after conditioning were used for subsequent procedures and testing.

### 2.2. Evaluation of Parameters Influencing the KA Oxidation Signal and Sensor Optimization Protocol

The influence of certain parameters, specifically the number of composite layers used for carbon working electrode (WE) functionalization and the reduction of GO, a component of the composite suspension, on the KA oxidation signal was evaluated. The sensor development protocol was subsequently adapted based on the optimal parameters obtained.

#### 2.2.1. Influence of Functionalization Parameters—Number of Suspension Layers

**Figure 1:**
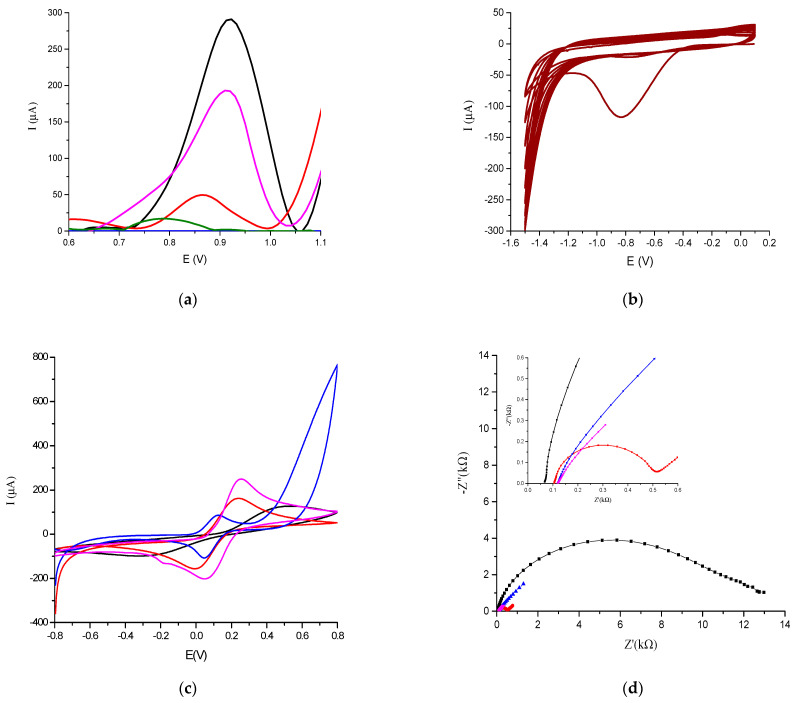
(**a**) DPVs obtained for a carbon-printed platform functionalized with different composite layers, i.e., three layers (black), two layers (purple), and one layer (red), in the presence of 500 µM KA solution prepared in the B–R buffer (pH 7), compared to the unfunctionalized platform in the B–R buffer (blue) and 500 µM KA in the B–R buffer (green). (**b**) CVs recorded during the electrochemical reduction of GO to rGO for 10 cycles between 0.2 V and −1.5 V. CVs (**c**) and Nyquist plots of EIS (**d**) recorded in the presence of 5 mM K_4_[Fe(CN)_6_]/K_3_[Fe(CN)_6_] in 0.1 M KCl for unmodified carbon-based electrodes (black), after pretreatment (orange), composite film-functionalized electrodes (PEI/AuNPs/GO) before GO reduction (blue), and after reduction (purple). Inset: magnified high-frequency region for the EIS spectra.

The intensity of the electrochemical response was assessed based on the application of one, two, or three layers of composite suspension for immobilization, with solvent evaporation between two successive depositions. The platforms obtained after functionalization were tested using the DPV method, and the resulted voltammograms recorded in the presence of 500 μM KA solution in Britton–Robinson (B–R) buffer of pH 7 are shown in Figure 1a.

An increase in the KA oxidation signal intensity was observed with the increasing number of polymeric layers applied. Comparing the signal intensities, a fourfold increase (I_ox_ = 180.10 μA) was observed for two composite layers and a sixfold increase (I_ox_ = 287.34 μA) for three layers compared to a single drop (I_ox_ = 46.80 μA). For the unfunctionalized carbon electrode, I_ox_ was only 8.64 μA, highlighting the electrocatalytic effect of the composite film. Therefore, the subsequent protocol was adapted to include the functionalization of the electrochemical platform with three composite film layers of PEI, GO, and AuNPs.

#### 2.2.2. Evaluation of the KA Signal Intensity Dependence on Oxidized or Reduced GO

GO reduction can be achieved by chemical (using reducing agents), thermal, photocatalytic, or electrochemical methods. Among these, electrochemical reduction is the most advantageous in terms of toxicity, cost, and experimental conditions. Additionally, electrochemically reduced GO (rGO) retains several properties of GO while improving others, particularly electrical conductivity.

Electrochemical reduction of GO was performed using an optimized CV procedure (10 cycles) in phosphate buffer saline (PBS) (pH 7). The process, illustrated in Figure 1b, showed an irreversible reduction signal at approximately −0.8 V which significantly diminished after the first cycle. Signal stabilization in the voltammogram suggests successful GO reduction to rGO, consistent with prior studies [36], and a stable sensor surface was achieved.

To evaluate the properties imparted by electrochemical GO reduction and to compare the electrochemical characteristics of modified and unmodified platforms post-immobilization, CV and EIS tests were performed in the presence of a redox probe ([Fe(CN)_6_]^3−^/^4−^, 5 mM in 0.1 M KCl). The goal was to evaluate the working electrode surface properties regarding electron transfer rates before and after GO reduction.

The resulting cyclic voltammograms (Figure 1c) reveal a decrease in electron transfer rate following WE surface functionalization with the composite film. The oxidation/reduction current intensities decreased from 123.5/−97.1 μA for the unmodified carbon electrode (black) and 160.4/−158.9 μA after activation (orange) to 82.7/−104.25 μA (red), attributed to the insulating effect of GO in the composite film.

However, significant increases in oxidation/reduction currents were observed after electrochemical reduction, with intensities reaching values of 253.5/−202.1 μA (blue). Post-reduction, the peak separation of the redox probe narrowed to less than 0.1 V, indicating successful electrode functionalization and improved conductivity, transforming GO from an insulator to a conductor, these results being consistent with prior reports [36,37].

The EIS results (Figure 1d) support these findings. The Nyquist diagrams of the unmodified electrode show an R_ct_ of ~11.4 kΩ, indicating slow electron transfer at the electrode surface. After electrochemical cleaning, R_ct_ decreased to 383 Ω, and, after composite film functionalization, R_ct_ increased to 816 Ω, confirming the weak conductivity of GO. The electrochemical reduction of GO resulted in a significant decrease in R_ct_ to approximately 64 Ω, consistent with CV data, demonstrating the successful transformation of GO into a highly conductive material.

The Nyquist spectra also show a well-defined diffusion component at the electrode interface after GO reduction, indicating faster electron transfer. The Nova 1.10.4 software was used to model the WE processes before and after functionalization. The equivalent circuit was identified as [R_s_(CPE[R_ct_W])] before GO reduction, including R_s_ (solution resistance), R_ct_ (charge transfer resistance), W (diffusion component), and CPE (constant phase element), replacing capacitance for porous/multiphase platforms.

Post-reduction, the circuit evolved to [R_s_(CPE[R_ct_W])(R_1_C_1_)], indicating a change in the transfer mechanism during sensor development. GO reduction enhanced electron transfer rates and surface conductivity. The small chi-square (χ^2^) values for the Pearson statistical test (Table 1) indicated a favorable fit between experimental data and the proposed circuit model [38,39].

As observed from the CV and EIS data, the peak oxidation/reduction current intensities were significantly higher after GO reduction, while the charge transfer resistance was notably greater for the platform where GO was not reduced. This confirms the superior conductivity of rGO and the improved analytical performance of the platform after reduction. Consequently, the GO reduction step was performed after each composite immobilization and prior to testing.

The electroactive surface area of the WE after the functionalization with the composite film and electrochemical reduction of GO was evaluated by analyzing the behavior of 5 mM [Fe(CN)_6_]^3−/4−^ used as a redox probe at different scan rates. Additionally, the Randles–Sevcik equation [40], presented below, was applied to the CV data:

$$I_{p}=(2.69\cdot 10^{5})\cdot n\cdot \sqrt{\alpha \cdot n_{\alpha }}\cdot C\cdot \sqrt{D}\cdot \sqrt{v}\cdot S$$

The parameters included in this equation are: the peak current intensity—I_p_ (A); the number of electrons transfered at the electrode—n (one in the case of this redox probe); the transfer coefficient—α; the molar concentration of the redox probe—C (5 × 10^−3^ M); the diffusion coefficient—D (cm^2^/s); the scan rate—v (V/s); S, which represents the surface area of the working electrode (cm^2^).

After functionalization, the electrode exhibited an electroactive area of 0.786 cm^2^, significantly larger than the unmodified electrode’s 0.126 cm^2^. This increased area accounts for enhanced electrochemical signals observed in CV and EIS results, as depicted in Figure 1a,c,d. Thus, the functionalized surface enhances electron transfer and the overall sensitivity for KA electrochemical direct detection.

### 2.3. Morphological, Topographical, and Elemental Characterization of the Platform Used for KA Detection

**Figure 2:**
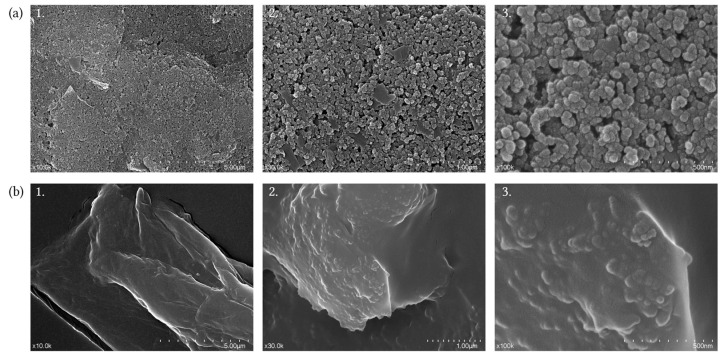
Comparative SEM images are presented for: (**a**) the unmodified carbon-based electrode surface; (**b**) the carbon electrode surface modified with the PEI/AuNPs/GO composite at different magnifications (scale bars: 5 μm (1); 1 μm (2); 500 nm (3)).

To enable a comparative analysis of the two electrochemical surfaces used in this study, SEM was employed as an initial characterization technique. SEM was applied to examine the surfaces before and after functionalization with the composite film of PEI/AuNPs/GO, with the results shown in Figure 2. The SEM images reveal the successful coverage of the carbon WE with the composite film, highlighting GO sheets (Figure 2(b1)) and nanostructures that can include both carbon nanoparticles in the substrate and AuNPs in the composite film embedded in the polymer (Figure 2(b3)).

**Figure 3:**
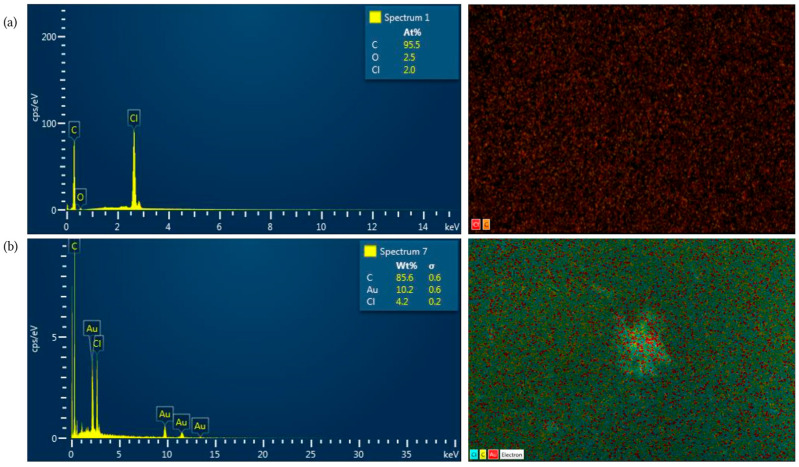
EDX spectra provide elemental composition data. On the left, the EDX spectra illustrate the elemental profile of the samples, while, on the right, the corresponding elemental distribution maps are shown. (**a**) The unmodified carbon-based electrode surface; (**b**) the carbon electrode surface modified with the PEI/AuNPs/GO composite. Scale bar: 500 nm.

Additionally, energy dispersive X-ray spectroscopy (EDX) was performed on the same electrode surfaces to analyze their elemental composition. Comparative results for the two electrode surfaces are shown in Figure 3. The presence of gold, indicating the successful immobilization of the composite on the WE surface, is evident in both the spectra and the elemental distribution maps. A gold content of 10.2% (±0.6) was detected (Figure 3b).

**Figure 4:**
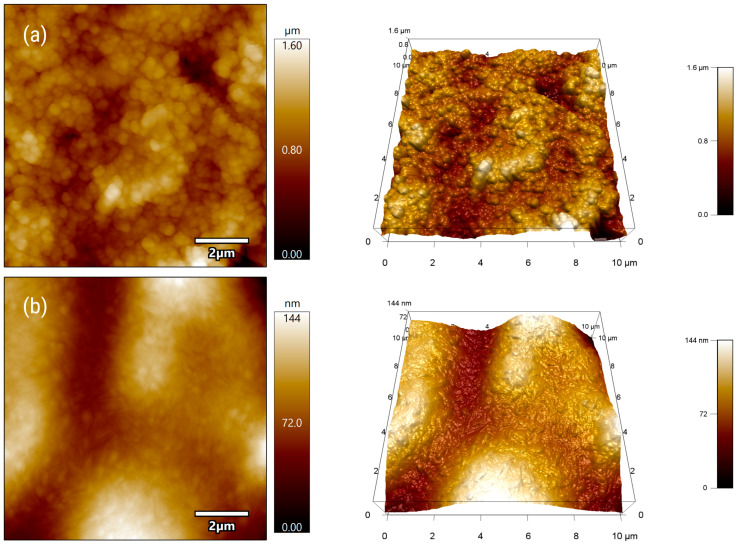
2D (**left**) and 3D (**right**) topographic AFM images of the unmodified electrochemical surface (**a**) and of the surface modified with a composite film (**b**). Scan size 10 µm.

The next step in the electrode surface characterization involved AFM for morphological and topographical analysis of the electrochemical sensor. AFM data confirmed the observations from SEM, showing the structure of the bare carbon electrode consisting of nanostructures distributed over the entire surface (Figure 4a), often overlapping in agglomerations. After modification, the presence of a PEI/AuNPs/GO nanocomposite film on the electrode surface considerably changed the surface structure of the bare carbon electrode (Figure 4b), smoothing the surface and decreasing its roughness; also, the maximum height of a 10 µm scan size decreased from 1.6 µm for the bare carbon electrode (Figure 4a) to 144 nm for the PEI/AuNPs/GO-modified electrode (Figure 4b). For the modified electrode, agglomerations were not well resolved, and the nanostructures were no longer well defined, since the PEI matrix uniformly covered the bare carbon electrode surface and embedded AuNPs and GO in the composite film.

### 2.4. Study of the Influence of Electrolyte pH

**Figure 5:**
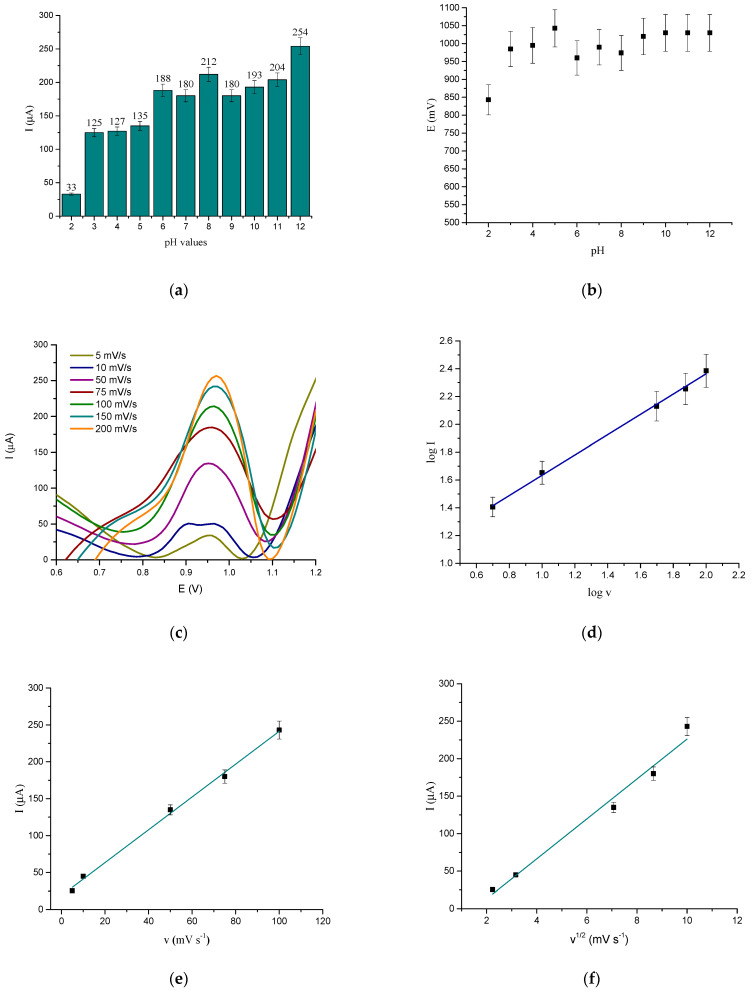
(**a**) Dependence of the oxidation current intensity of KA on the pH values of the electrolytic medium obtained for 75 μM KA in the B–R buffer at different pH values in the range of 2 to 12 (bar chart—oxidation current values are displayed above each column, with error bars representing standard deviation values relative to the mean intensity value for each signal. (**b**) Variation of the oxidation peak potential of KA 100 μM with the pH values of the electrolyte medium—B–R buffer at different pH values (the tests were performed in triplicates, the corresponding mean is presented, and the error bars represent the standard deviation in relation to the mean corresponding to each pH value). (**c**) Overlay of DPVs obtained for 100 μM KA in the B–R buffer at pH 7, recorded at different scan rates. (**d**) Variation of the logarithm of the oxidation peak current with the logarithm of the scan rate for the electrochemical transformation of 200 μM KA, using scan rates ranging from 5 to 200 mV s^−1^ (the graph includes the linear equation and error bars). Variation of the oxidation peak current intensity with the scan rate (**e**) and the square root of the scan rate (**f**) for the electrochemical transformation of 75 μM KA (the graph includes error bars, estimated as the standard deviation relative to the mean current values obtained from three individual measurements for each tested scan rate).

Considering the chemical structure of KA, which suggests a shift in the mechanism of reaction with changes in the pH values of the testing medium, a study was conducted to examine the influence of this parameter on the DPV signal obtained for the analyte. The electrochemical behavior of 75 µM KA solutions was analyzed across a pH range from 2 to 12 using the optimized DPV technique and the B–R buffer of different pH values. It was observed that, in the PBS medium at pH 7, differential pulse voltammograms obtained for the electrochemical oxidation of KA within the 0.5–1.4 V potential range revealed two distinct peaks: one at about 0.9 V and another around 1.15 V, a phenomenon reported in another study [41]. Optimization tests employed the B–R buffer due to its broader pH testing range compared to PBS. In the B–R buffer, both signals were clearly observed under specific scan rates and pH conditions, as illustrated, for example, by the blue curve in Figure 5c. The consistently visible second peak at 1.15 V was used for data collection and representation throughout the manuscript, the first one [41]. Figure 5a reveals that the oxidation current intensity increased sharply from pH 2 to pH 3, remained relatively constant until pH 5, increased again until pH 8, and then exhibited a fluctuating trend in the alkaline range, with the highest value observed at pH 12. This behavior suggests the involvement of protons in the oxidation mechanism, as reported before [34,41,42]. Regarding the mechanism for the electrochemical transformation of KA, the limited available literature indicates a reversible oxidation/reduction signal at about 0.3 V. Thus, Bornaei et al. [34] proposed a reversible KA detection using metal–organic frameworks, highlighting a mechanism involving equal proton–electron participation under highly acidic conditions. Similarly, Poursaeed et al. [35] identified a reversible signal near 0.3 V in neutral PBS but did not propose a specific mechanism. Our study identified a distinct oxidation signal at about 1.15 V which is likely associated with the oxidation of the 4-hydroxyl group, consistent with L. Kubikova’s findings [41]. This is because the *p*-hydroxy-imino group in KA’s structure is much more stable than the o-hydroxyl group [34]. Additionally, an anodic shift in the oxidation signals of KA with increasing pH was observed (Figure 5b). This phenomenon can be attributed either to the involvement of protons in the electrochemical transformation mechanism or to the reduced stability and solubility of KA at lower pH values. This observation aligns with literature data indicating that KA has good solubility at pH 7, while its solubility decreases significantly in the pH range of 3–4 [43]. It has also been reported that the pKa of the *o*-hydroxyl group is 10.5, while the pKa for the nitrogen atom in the heterocyclic ring is 3.5 [34].

All tests were performed in triplicates, with mean values presented and error bars indicating the standard deviation (SD) relative to the mean peak height obtained for each tested pH value. As shown in Figure 5a, the intensity value obtained for 75 µM KA in the B–R buffer at pH 7 was 180 µA, which is satisfactory considering the project’s purpose to quantify the biomarker in saliva, where physiological pH values range between 6.7 and 7.3.

### 2.5. Study of the Influence of the Scan Rate

The influence of the scan rate on the electrochemical oxidation of KA was tested in 0.1 M B–R buffer at pH 7. This medium was selected to simulate the environment in which the sensor would be used. The DPV results for scan rates ranging from 5 to 200 mV/s are shown in Figure 5c. An increase in the scan rate led to a rise in the oxidation peak current for KA, accompanied by a slight anodic shift to higher potentials (Figure 5c). A scan rate of 100 mV s^−1^ was selected for subsequent experiments, as it provided a sufficiently high current intensity while remaining slow enough to capture the electrochemical transformation of KA.

The variation in oxidation signal intensity with the scan rate (Figure 5e) and with the square root of the scan rate (Figure 5f) was analyzed to evaluate the kinetics of the electro-oxidation process for KA. The relationship between the oxidation current intensity (I_ox_) and the scan rate is described by the equation: I_ox_ (μA) = 2.22 v (mV s^−1^) + 19.01. For the variation of the oxidation current with the square root of the scan rate, the equation is: I_ox_ (μA) = 26.62 v^1/2^ (mV s^−1^)^1/2^ – 40. A stronger linear correlation was observed for the relationship between oxidation current intensity and scan rate (R^2^ = 0.997) compared to its variation with the square root of the scan rate (R^2^ = 0.982). This indicates an adsorption-controlled process.

This finding contrasts with previously published results, which identified a diffusion-controlled behavior for KA oxidation on carbon paste electrodes modified with terbium-doped zinc oxide nanostructures [35] and on carbon paste electrodes modified with chromium-based metal–organic frameworks (MOFs) [34].

To better understand the kinetics of the electro-oxidation process, a graph of the logarithm of the oxidation current intensity versus the logarithm of the scan rate was plotted (Figure 5d). The resulting linear correlation is described by the equation: log(I_ox_ (μA)) = 0.73 log(v (mV s^−1^)) + 0.90. The slope of 0.73 is close to both the theoretical value of 0.5, characteristic of diffusion-controlled processes, and the theoretical value of 1, characteristic of adsorption-controlled processes. These results align with previously published data [34,35] and suggest that the overall electro-oxidation process of KA on the carbon-based sensor functionalized with the PEI/AuNPs/rGO composite film is controlled by both diffusion and adsorption. This phenomenon may be attributed to the nature of the in-lab printed electrode surface, which may exhibit roughness and irregularities.

### 2.6. Analytical Parameters of the Electrochemical Sensor

After the electrochemical characterization of the nanostructured platform, the electrochemical fingerprint of KA was established, combining the experimental conditions that produce the highest oxidation signal and maximum detection sensitivity.

CV tests performed in the PBS medium with a pH of 7 yielded a low-intensity reversible signal but revealed an additional oxidation signal near 1 V, dependent on KA concentration. In PBS of pH 7, it was observed that the differential pulse voltammograms obtained for the electrochemical oxidation of KA in the potential range from 0.5 V to 1.4 V showed two peaks, one at approximately 0.9 V and the second at approximately 1.15 V. A similar pattern was reported by Kubikova et al. [41], who observed two DPV peaks at approximately 1.020 V and 1.145 V, corresponding to KA oxidation. These authors suggested that the first peak might result from the oxidation of the heterocyclic NH group, potentially linked to the formation of a stable keto tautomer, while the second one at higher potential results from the oxidation of the 4-hydroxy group. This aligns with the shoulder-like peak we observed, supporting its attribution to a similar electrochemical transformation.

The optimized analysis parameters chosen were a scan rate of 100 mV s^−1^ and a PBS solution of pH 7, which is close to the physiological pH of saliva and corresponds to the maximum oxidation signal intensity of the (bio)marker.

**Figure 6:**
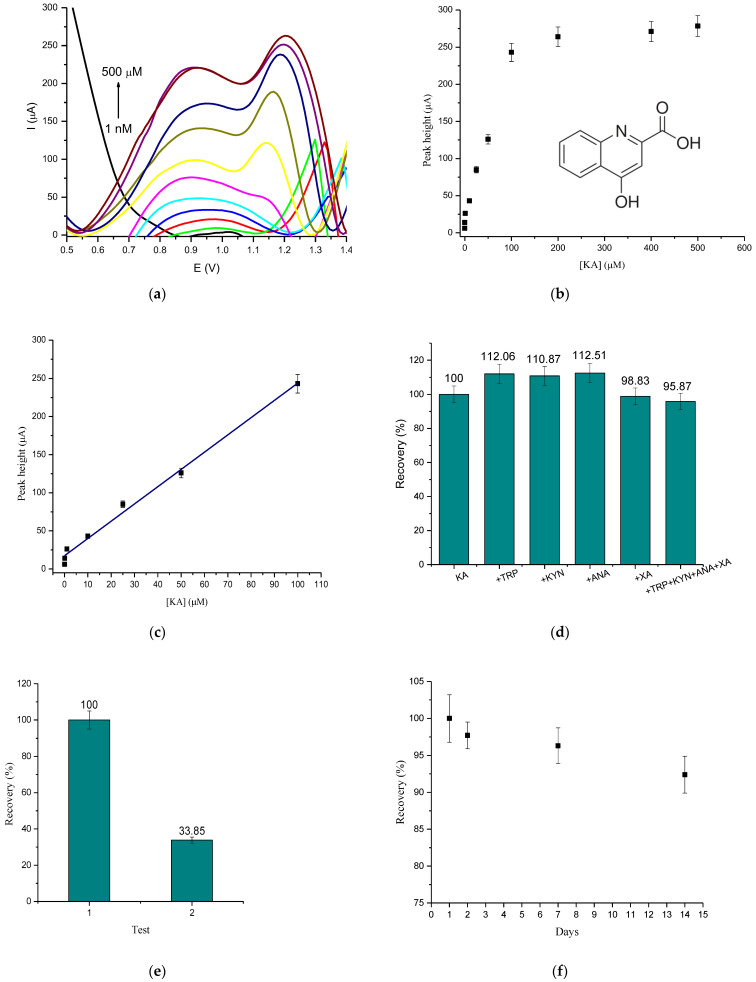
(**a**) Overlay of voltammograms corresponding to different concentrations of KA (1 nM, 10 nM, 100 nM, 1 µM, 10 µM, 25 µM, 100 µM, 200 µM, 400 µM, 500 µM) prepared in 0.1 M PBS buffer, pH 7. The test conducted in the buffer using the optimized sensor is represented in black). (**b**) Logarithmic dependence of the oxidation peak current intensity on the KA concentration. (**c**) Calibration curve illustrating the linear dependence of the oxidation current intensity on KA concentration (within the range of 1 nM–100 µM) obtained via DPV (tests were performed in triplicates). (**d**) Recoveries calculated for the KA signal following a selectivity study conducted in PBS buffer at pH 7 with (TRP)and other metabolites from the kynurenine pathway (bar chart—recovery percentages are displayed above each column, with error bars representing standard deviation values relative to the mean recovery for each signal). (**e**) Recoveries calculated for the KA signal following intra-assay stability testing. (**f**) Long-term stability study of the PEI/AuNPs/GO platform. KA signal recovery tested via DPV on days 1, 2, 7, and 14.

DPVs recorded using the carbon-based sensor functionalized with the PEI/AuNPs/rGO composite film for KA solutions of different concentrations from 1 nM to 500 µM, are presented in Figure 6a. At low KA concentrations (from 1 nM to 10 µM), the voltammograms display a single peak, whose intensity increases with concentration, while, at higher concentrations (from 25 µM to 500 µM), the voltammogram shape changes, showing the appearance of two peaks. From Figure 6b, the DPV results indicate a logarithmic relationship between the oxidation current and the KA concentration across the 0.001–500 µM range. However, within the 1 nM to 100 µM range, the variation is linear (Figure 6c) and is described by the equation: I_ox_ (μA) = 2.33 ⋅ [KA] (μM) + 12.29; (R^2^ = 0.9921). The limit of detection (LOD) was estimated at 0.3 nM, and the limit of quantification (LOQ) was 1 nM, based on a signal-to-noise ratio of 3 for LOD and 10 for LOQ, respectively (based on a minimum of three repetitions for each parameter). The relative standard deviation (RSD) was calculated for peak heights obtained from at least three repetitions per concentration, across the tested KA concentrations, then the arithmetic mean of individual RSD values was determined as 3.85%. LOQ was set to the lowest tested concentration, 1 nM, and the sensitivity was 2.33 µA µM^−1^. The obtained parameters were compared with previously published data (Table 2). 

The developed sensor for KA direct detection offers a significant advancement in sensitivity and reliability compared to previously reported methods. With a limit of detection (LOD) of 0.3 nM and a linear range of 1 nM to 100 µM, it matches or outperforms most alternative approaches, except for those using biocomponents like antibodies based on indirect detection. Unlike biocomponent-based systems, this sensor relies on a robust, direct electrochemical detection approach, minimizing complexity and cost. Its flexible platform, using PEI/AuNPs/rGO-functionalized electrodes, provides exceptional repeatability (RSD = 3.85%) and usability with real biological samples, such as saliva and serum, as well as the validation compared against an optimized HPLC method.

Alongside testing KA solutions of varying concentrations on the functionalized composite film platform, tests were also conducted on the unmodified carbon platform. A comparison of the peak intensity values obtained for the same concentrations on the two electrochemical platforms revealed significant increases for the nanostructured platform. These observations confirm the utility of the composite film deposited on the carbon electrode for the direct electrochemical detection of KA. For the unmodified carbon platform, a linear relationship between the oxidation current and the KA concentration was observed in the range of 25–500 µM, described by the equation: I_ox_ (μA) = 0.017 ⋅ [KA] (μM) + 0.1374; (R^2^ = 0.9972). The sensitivity was determined to be 0.017 µA µM^−1^. Thus, it can be noticed that the functionalization with the nanocomposite film resulted in an approximately 134-fold improvement in sensitivity for KA detection and extended the concentration range by four orders of magnitude, underscoring the necessity of electrode functionalization.

### 2.7. Selectivity Study

The selectivity of the carbon-based sensor functionalized with the PEI/AuNPs/rGO composite film for KA detection was also evaluated. A literature review was conducted to identify potential interferents for this biomarker. Other metabolites from the kynurenine pathway—the degradative pathway of TRP, which also produces KA—were chosen due to their structural similarities with the biomarker. The following compounds were tested: TRP; KYN; ANA; XA. These compounds are biologically relevant and occur naturally in the body. To assess the electrochemical sensor’s selectivity for KA, physiological concentrations of the interferents were used: TRP: 60 µM; KYN: 2 µM; ANA: 0.13 µM; XA: 25 µM, while the KA concentration was set to 100 µM. Tests were conducted in 0.1 M PBS buffer at pH 7 using the optimized DPV procedure. The recovery rates of KA were evaluated both in mixtures containing KA and a single interferent, as well as in a multicomponent mixture including the analyte and all interferents from the study (Figure 6d).

### 2.8. Reproducibility and Stability of the Sensor

To evaluate the reproducibility of the PEI/AuNPs/rGO nanocomposite film deposition on lab-printed electrodes, five electrodes were prepared using the same optimized procedure described before. These electrodes were used to assess the DPV signal corresponding to the electrochemical oxidation of KA (50 µM in 0.1 M PBS, pH 7). The average RSD for the peak current values was calculated to be 4.76%, indicating good reproducibility for KA determination.

The intra-analysis stability of the sensor was tested by performing repeated measurements on the same electrode, each time using a new volume of a standard solution of 50 µM KA. Between tests, the electrode was thoroughly rinsed with ultrapure water. The recovery values, calculated relative to the peak current intensity recorded in the first test, are shown in Figure 6e for the first two tests only. A significant decrease was observed, with a recovery rate of only 34% in the second test. This decline prevents sensor reuse. However, this limitation is not considered a disadvantage given the sensor’s low cost and its design for single-use applications, which eliminate the risk of contamination in real biological fluid samples.

The sensor’s stability over time was assessed by testing sensors prepared on the same day and stored under clean and dry conditions at room temperature. These sensors were tested after 1, 2, 7, and 14 days in the presence of 50 µM KA. Results demonstrated excellent signal recovery compared to the initial day, as illustrated in Figure 6f. This indicates that the platform remains stable for at least 14 days after preparation and can reliably be used for KA detection during this period.

### 2.9. Testing the Electrochemical Sensor on Biological Samples

The final objective was to validate the electrochemical sensor by testing it on biological samples to assess its suitability for detecting KA in complex matrices. Initially, the electrochemical response of KA was evaluated in an artificial matrix—simulated saliva. Following this step, the sensor was tested on human saliva samples collected from a healthy volunteer. The samples were spiked with KA at the following concentrations: 25 µM, 50 µM, and 100 µM. The tests were conducted using the optimized DPV procedure established before.

The sensor’s performance was further evaluated in real biological samples, including commercial physiological saline and human saliva. For human saliva, samples were collected from healthy volunteers using Salivette^®^ (SARSTEDT, Nümbrecht, Germany), sterile plastic containers. Patient consent was obtained prior to collection, and all institutional recommendations for handling human-derived samples were followed, as approved by the Ethics Committee of UMF Cluj-Napoca (Approval No. 117/17.05.2022). To enhance compatibility with the sensor, saliva samples were also tested after dilution in a 1:1 ratio with 0.1. M PBS pH 7. The preparation and testing protocol for these diluted samples was identical to that for artificial saliva. The recovery rate of KA in these biological samples was assessed and is summarized in Table 3. These results provide a quantitative measure of the sensor’s accuracy and efficacy in detecting KA in both artificial and real saliva matrices.

The results demonstrate that the recovery rates obtained were satisfactory, ranging between 88.68% and 115.79% for the current intensity, corresponding to the oxidation peak of KA. This indicates that the biological matrices tested—serum and saliva—did not interfere with the quantification of KA.

To further validate the results obtained from the direct electrochemical detection of KA, raw saliva samples from 15 participants in the study were analyzed using the optimized DPV method and a HPLC-UV protocol as a control method. For the HPLC-UV method, the calibration curve was constructed using KA standard solutions prepared in 0.1 M PBS at pH 7. Dilutions in the mobile phase produced solutions ranging from 0.1 nM to 100 µM. The calibration curve demonstrated a strong linear relationship over the tested concentration range, described by the equation: peak area = 75,269 ⋅ [KA (µM)] + 58,412; R^2^ = 0.9994. The LOD was estimated at 30 pM for the control method based on a signal-to-noise ratio of 3, with an RSD value of 2.54% across the tested KA concentrations.

As shown in Table 4, the KA concentration was significantly lower in the control group compared to the patient group. The average KA concentration for the control group was 7.25 nM, consistent with physiological salivary levels reported in the literature, whereas the patient group had an average KA concentration of 47.50 nM, indicating a potential association with inflammatory conditions. The recovery rates between 92.77% and 112.76% calculated based on the electrochemical detection results were successfully validated using the HPLC-UV as a control method.

### 2.10. Robustness of the Electrochemical Method

The reliability of the electrochemical method for detecting KA in raw, unprocessed saliva from patients and controls (intra-assay validation of the optimized DPV method) was also assessed in this study. Saliva samples from all participants, namely 10 patients and five controls, were analyzed using both the electrochemical method and the HPLC-UV protocol, with three replicates performed for each measurement. An ANOVA regression analysis of the results showed a high level of statistical significance, with no deviations observed in parallelism or linearity (*p* = 0.976 > p_theoretical_ = 0.05). All assays produced results within the confidence interval, indicating accurate implementation of the analysis system. Furthermore, the DPV method demonstrated excellent sensitivity and a distinct response compared to HPLC-UV-derived concentrations, highlighting its robustness and reliability for KA detection. These findings, summarized in Table 5, underscore the capability of the DPV method as a reliable tool for analyzing KA levels in biological matrices such as saliva.

**Figure 7:**
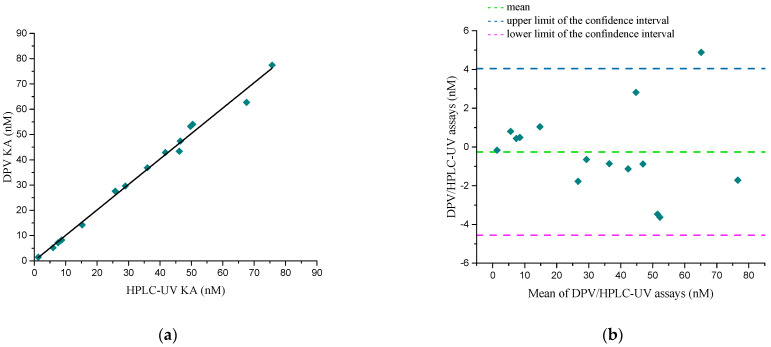
(**a**) The correlation between KA concentrations in human saliva, measured using DPV and the HPLC-UV control method, analyzed for 10 patients and five controls (n = 15). (**b**) The Bland–Altman plot evaluating the agreement between the two methods. Each point represents the difference in KA concentrations (nM) measured by DPV and HPLC-UV relative to their mean. The horizontal green line shows a mean bias of −0.254 nM KA, while the dashed purple and blue lines indicate the 95% confidence interval (±2.19 nM), highlighting a strong agreement between the two methods.

Validation of the DPV method was obtained using the optimized HPLC-UV as a control method. Both techniques analyzed raw saliva samples collected from 10 patients and five controls, each containing unknown concentrations of KA, with three replicates per sample. A statistical comparison using a two-sample *t*-test assuming equal variances showed no significant differences between the results of the two methods (*p* = 0.456, where *p* > 0.05). Furthermore, a strong correlation was observed between KA levels obtained through the DPV and the HPLC-UV assays. The statistical evaluation of recovery data obtained for raw saliva samples was performed using the optimized DPV electrochemical sensor in comparison to the reference HPLC-UV method. The analysis demonstrated a strong correlation, with no statistically significant differences between the results obtained by the two methods (Figure 7a). This was confirmed by an excellent regression coefficient of 0.9911 (*p* < 0.001) and a slope of 1.007, suggesting that both methods are highly consistent in their performance for KA determination.

To further assess the consistency between the methods, a Bland–Altman plot was generated (Figure 7b). This plot compared the differences in KA concentrations between the methods against the mean values. The mean difference was −0.2538 nM (green dashed line), with limits of agreement ranging from −4.01 (purple dashed line) to 4.8 nM (blue dashed line). Importantly, the majority of the differences fell within these limits, signifying a strong agreement.

The Bland–Altman plot also highlighted the accuracy of the DPV method, as the observed mean difference was minimal and within acceptable boundaries for analytical accuracy. These findings underscore the robustness and reliability of the DPV method for monitoring KA concentrations in human saliva. The high degree of agreement and the excellent correlation between the two techniques validate the DPV method as a suitable alternative to HPLC-UV for KA analysis. This optimized electrochemical detection strategy offers a precise, reliable, and potentially more accessible approach to saliva-based KA monitoring in biological samples.

## 3. Materials and Methods

The following reagents were used in this study: potassium hexacyanoferrate(III) (K_3_[Fe(CN)_6_]), potassium hexacyanoferrate(II) (K_4_[Fe(CN)_6_]), potassium chloride (KCl), sodium chloride (NaCl), monosodium phosphate (NaH_2_PO_4_), disodium phosphate (Na_2_HPO_4_), hydrochloric acid (HCl), sulfuric acid (H_2_SO_4_), boric acid (B(OH)_3_), phosphoric acid (H_3_PO_4_), acetic acid (CH_3_COOH), sodium hydroxide (NaOH), TRIS (tris(hydroxymethyl)aminomethane—(HOCH_2_)_3_CNH_2_), kynurenic acid (4-Hydroxyquinoline-2-carboxylic acid), polyethyleneimine in aqueous suspension of 50% concentration (ρ = 1.08 g mL^−1^). These reagents were purchased from Sigma Aldrich (St. Louis, MO, USA).

To modify the electrochemical surface, a gold nanoparticle solution with a size of 15 nm (0.05 mg mL^−1^—AuNPs (Nanovex Biotechnologies, Asturias, Spain)) was used alongside solid graphene oxide—GO (Sigma Aldrich, St. Louis, MO, USA). For selectivity studies, TRP, KYN, ANA, and XA were used, all purchased from Merck (Merck, Rahway, NJ, USA), while, for studies on real samples, artificial saliva and human serum were used (Sigma Aldrich, St. Louis, USA), alongside human saliva collected from healthy volunteers.

The collection of the human saliva was performed from healthy volunteers using sterile Salivette^®^-type plastic containers (Sarstedt Group, Nümbrecht, Germany). The patients’ consent was obtained before sampling and all institutional recommendations regarding the manipulation of real samples were followed (with the consent of the Ethics Commission of UMF Cluj-Napoca No. 117/17.05.2022). The collection devices were equipped with an absorbent cotton pad, which allows the collection of volumes between 0.8–1.4 mL. Saliva was collected at least 60 min after the ingestion of food (solid or liquid), the administration of certain drugs, or the performance of oral hygiene in order to avoid contamination of the sample with interferents. The absorbent pad was removed from the collection device and placed in the oral cavity for 2 min without chewing. Afterwards, the swab was inserted into the collection device, the exact time of sample collection was noted on the device, and then the device was sealed and centrifuged for 2 min at 2000 rpm. To avoid bacterial contamination, the sample was stored at −20 °C until use. No other pretreatments were applied to the saliva samples before the tests were performed.

For the electrochemical characterization of the electrode surfaces before and after each functionalization step, an equimolar solution of 5 mM potassium ferro-ferricyanide (K_4_[Fe(CN)_6_]/K_3_[Fe(CN)_6_]) in KCl 0.1 M was used. To evaluate the behavior of the sensor in the presence of KA at different pH values of the solutions, a B–R buffer solution was prepared which contained equal concentrations of 40 mM boric acid, phosphoric acid, and acetic acid. The pH adjustment for the B–R buffer was conducted with HCl and NaOH using a VioLab 50 Basic pH meter (The Carl Roth GmbH + Co. KG, Karlsruhe, Germany).

PBS of pH = 7 was prepared using equal concentrations (0.1 M) of NaH_2_PO_4_, Na_2_HPO_4_, and 100 mM NaOH, and the pH value was adjusted with HCl and NaOH (Sigma Aldrich, St. Louis, USA) using the aforementioned pH meter.

### 3.1. Electrochemical Methods

The electrochemical methods used in this study were CV, DPV, and EIS. By using these techniques, the modifications found on the surface of the electrode were monitored in different steps of the study: optimization, actual detection of the analyte, and in the assessment of the feasibility of using the biosensor in complex biological matrices which contain analytes that can not only constitute interferences for the detection of KA, but also have a matrix effect through non-specific adsorption phenomena, blocking or creating an impediment for electron transfer.

Two CV methods were optimized for evaluating the electrochemical properties of the platform after the functionalization of carbon electrodes with a different number of composite layers, respectively, after the GO reduction step. In the first case, a potential window of −0.5 V to +0.8 V was used, with a scan rate value of 0.1 V s^−1^, a potential step of 0.00244 V for two cycles, in the presence of an equimolar solution of [Fe(CN)_6_]^4−/3−^ 5 mM in 0.1 M KCl.

For the electrochemical reduction of GO to rGO, CV was used with a potential range from +0.1 V to −1.5 V, a scan rate value of 0.1 V s^−1^, and a potential step of −0.00244 V for 10 cycles for the electrochemical reduction of polymer-immobilized graphene oxide on the surface of the working electrode.

DPV tests were performed in the potential range from −1.0 V to +1.4 V with a scan rate value of 0.1 V s^−1^ and a potential step of 0.01 V. An equilibration time of 30 s was applied and the technique was optimized; therefore, it was used for the actual quantification of KA, for the evaluation of the signals obtained depending on the pH values of the electrolytic medium, for the quantification of the analyte in complex matrices, and in the presence of interfering metabolites of the kynurenine pathway. The influence of the variation of the scan rate on the intensity of the KA oxidation signal was also evaluated by this method.

EIS tests were performed at a frequency range from 0.1 to 10,000 Hz, applying 61 frequencies with an amplitude of 0.01 Hz to evaluate the variations in charge transfer resistance with surface functionalization with the composite film, but also after the reduction of GO to rGO to demonstrate the improvement of the conductive properties of the surface.

### 3.2. Morphological, Topographic, and Elemental Characterization of the Sensor Surface

The electrode surfaces were analyzed by scanning electron microscopy (SEM) before and after modification with the composite material, at different magnifications. Also, the same samples were analyzed with energy dispersive X-ray spectroscopy (EDX) and the elemental analysis was performed to evaluate the success of the functionalization. SEM images were obtained by using a Hitachi 8230 SEM at 30 kV, 10 µA, and a 14 mm working distance and EDS spectra were collected using an Oxford Instruments EDX detector and the AZtec 6.1SP1 Software.

Surface characterization in terms of morphology and topography was performed by atomic force microscopy (AFM), using a Cypher S microscope (Asylum Research, Santa Barbara, CA, USA). AFM images were obtained in air, under ambient conditions, in AC mode (tapping mode), using silicon cantilevers (AC160TS-R3, Olympus, Tokyo, Japan) with a typical spring constant of 26 N/m and a resonance frequency of 300 (±100) kHz. Data acquisition and image analysis were performed using the integrated AR16 software (Asylum Research) written in the Igor Pro software package (Igor Pro 6, WaveMetrics, Inc., Lake Oswego, OR, USA). Multiple areas of the sample surface were analyzed, with 512 pixels/line and with a scan rate of less than 1 Hz.

### 3.3. The Electrode Imprinting Process

The lab-made screen-printed electrodes used in this study were fabricated using special conductive and isolating inks, which were deposited on a plastic support via a stainless steel template with a cut-out pattern that mimicked the shape of the electrochemical cell and contacts. This stencil design ensured that the ink was evenly distributed in a layer of the same height. A rubber spatula was used to spread the ink onto the stencil. The three electrodes (working electrode (WE), reference electrode (RE), and auxiliary electrode (CE)) were printed by applying the same procedure but using different inks. A flexible plastic support was chosen for the electrochemical cell to withstand mechanical stress and to ensure the possibility of further development toward a wearable sensor, as has been done in other studies from our group [46,47,48]. The WE, representing the main electrode of the electrochemical cell, was made of carbon, as was the CE, and the RE was made of Ag. The area of the working electrode was about 50 mm^2^ and the size of the whole electrochemical sensor was 1 × 1 cm (length vs. width). The printing protocol consisted of five steps, as follows. First, the contacts for the three electrodes were printed using Ag ink, then, using the same ink, RE was printed. In the next step, CE was printed using carbon ink and the last electrode printed was WE using the same carbon ink. The final step consisted of printing an insulating layer over the contacts, which allowed the electrochemical cell to be isolated from the contacts and prevented shortcuts of the circuits when connected to the potentiostat. Each printed ink layer required a drying step at 50 °C for at least 15 min, and, after printing the isolation layer, the sensor was left for 30 min at room temperature. After drying, the ink film was 150 µm thick.

### 3.4. Elaboration of the PEI/AuNPs/GO-Based Sensor

A suspension containing polyethyleneimine (PEI—20 mg mL^−1^), gold nanoparticles (AuNPs—0.05 mg mL^−1^; 15 nm in diameter), and 5 mg of solid graphene oxide (GO) was prepared. The obtained suspension was homogenized for 30 min by ultrasonication, and, every 5 min, the container was additionally homogenized with a vortex system for 1 min.

Regarding the immobilization of the composite film on the WE surface, the suspension was homogenized for 30 min before pipetting and vortexed for 30 s between depositions to ensure homogeneity and reproducibility for the obtained surfaces. Between the composite layers, the electrochemical surfaces were dried under a stream of warm air and, at the end, were kept for 30 min in an oven at 50 °C to facilitate the evaporation of the solvent and the immobilization of the composite film on the electrode. The suspension was kept at 4 °C and was used for a maximum of two months.

### 3.5. Selectivity Study—Reproducibility and Stability of Sensor

The selectivity of the carbon-based sensor functionalized with a PEI/AuNPs/rGO composite film, developed for KA detection, was also evaluated. A literature review was conducted regarding potential interferents of this biomarker. It was decided to use other metabolites from the KP, the degradative pathway of TRP from which KA is also obtained, as these metabolites present structural similarities to the biomarker. The compounds used for this purpose were TRP, KYN, ANA, and XA (1), all of which are produced in the body together with the target analyte.

To assess the selectivity of the electrochemical sensor for the analyte, physiological concentrations were chosen for the interferents: 60 μM for TRP, 2 μM for KYN, 0.13 μM for ANA, and 25 μM for XA, while 100 nM was selected for KA. Tests were conducted in PBS at pH 7, using the optimized DPV procedure to evaluate the recovery rates of KA both from its mixture with a single interferent and from a multicomponent mixture containing the analyte and all the interferents included in the study.

### 3.6. Real Sample Collection, Preparation, and Testing

The analysis of real samples was carried out using spiked commercial human serum and human saliva collected from healthy volunteers. For saliva, undiluted samples were used, whereas human serum samples were diluted ten-fold with 20 mM PBS at pH 7.4. Both types of biological fluids were spiked with standard KA solutions to achieve target concentrations of 25 nM, 50 nM, and 100 nM prior to detection and quantification. To validate the performance of the developed sensor with real biological samples, the study included ten patients diagnosed with long COVID (confirmed by at least one positive COVID-19 test and a neurological evaluation) and five control subjects with no history of COVID-19 infection. This approach ensured a robust evaluation of the sensor’s accuracy and reliability in detecting KA in diverse biological matrices.

The study received approval from the Scientific Research Ethics Committee of the University of Medicine and Pharmacy “Iuliu Hațieganu” Cluj-Napoca, under approval number AVZ117, dated 17 May 2022. Participants were included in the study after they were informed about the study’s objectives and procedures, underwent a thorough medical evaluation, and signed the informed consent form and agreement for the processing and storage of personal data. Additionally, they completed a detailed questionnaire providing personal and medical information.

Saliva samples were collected using Salivette^®^ devices, which feature an absorbent cotton swab designed to collect 0.5–1.5 mL of saliva. To avoid contamination, samples were collected at least 60 min after the participants had eaten, drunk, taken medication, or performed oral hygiene. Participants were instructed to place the swab in their mouth for 2 min without chewing. After collection, the swab was returned to the device, sealed, and centrifuged for 2 min at 2000 rpm. The samples were then transported to the laboratory immediately after collection and stored at −20 °C to prevent bacterial growth.

For validation of the electrochemical detection results, high-performance liquid chromatography (HPLC) was employed using a ShimadzuCMB-20A HPLC system (Singapore), equipped with a Nucleosil C18 column (Allentown, PA, USA) and an SPA-10A VP UV-VIS detector (Singapore). The mobile phase consisted of 30% acetonitrile in ultrapure water, optimized for a flow rate of 0.4 mL min^−1^. KA detection was carried out at a wavelength of 216 nm, with a retention time of 1.78 min. All solvents used in the mobile phase were filtered through 0.22 μm cellulose filter membranes, and all sample solutions were filtered through 0.2 μm cellulose acetate membrane syringe filters to ensure purity and accuracy.

## 4. Conclusions

This study successfully demonstrates the development of a flexible and sensitive electrochemical sensor platform for the detection of kynurenic acid in biological samples. The integration of custom carbon-based electrodes with a composite film of gold nanoparticles, electrochemically reduced graphene oxide, and polyethyleneimine proved to be highly effective in enhancing the sensor’s conductivity and sensitivity. Characterization techniques confirmed the sensor’s structural and electrochemical properties, while the optimized DPV method yielded a broad dynamic range and a detection limit suitable for the determination of the target analyte in biological fluids.

The sensor offers significant advantages over traditional detection methods, including simplicity, cost-effectiveness, and rapid performance, without compromising sensitivity or accuracy. These findings underscore its potential utility as a reliable tool for kynurenic acid monitoring in clinical and research settings, particularly for addressing neurological and psychiatric disorders. Future work will explore the sensor’s applicability to other analytes and its scalability for broader diagnostic applications.

## Figures and Tables

**Table 1:** The equivalent circuit elements used for the fitting and simulation of the EIS data obtained during the optimization stages of the sensor platform.

Steps Involved in the Sensor Elaboration Protocol	R_S_ (Ω)	R_CT_ (Ω)	CPE (µA/V) N	W (mA/V)	R_1_ (Ω) C_1_ (mF)	χ^2^
Platform after printing	93	1140	1.56 0.91	0.86	- -	0.0062
Platform after activation	106	380	2.13 0.95	3.04	- -	0.0148
Platform functionalized with PEI/GO/AuNPs nanocomposite film	119	820	0.47 0.99	1.69	5.10 0.17	0.0632
Platform functionalized with PEI/rGO/AuNPs nanocomposite film	120	60	0.18 1.07	2.92	10.20 1.29	0.0044

**Table 2:** Comparison between the analytical parameters of the electrochemical sensor for KA developed in this study and other sensors in the literature.

Detection Method	Platform	LOD (nM)	Linear Range (nM)	Simultaneous Detection	Real Sample	References
CA, EIS	AuE/SAM/BSA-pseudo-KA/KA-Ab/Sec-Ab	0.39 0.28	0.01–0.10	-	Diluted serum	[31]
DPV	FL-NS Tb^3+^/ZnO/CPE	0.34	0.10–700,000	TRP	Serum, urine	[35]
DPV	MIL-101 (Cr)/CPE	17	100–150,000	TRP, KYN, XA, DP, GL, GLU, ASA	Diluted plasma 1:20	[34]
SWV	Nanocarbon film electrodes	400	-	TRP, KYN, 3-HK, ANA, 3-HAA	-	[44]
DPV	GO/chitosan	3.00	0.01–500	TRP, KYN, XA, DP, glucose, cortisol, ASA	Diluted plasma 1:10 human saliva	[45]
DPV	PEI/AuNPs/GO	0.3	0.001–100	TRP, KYN, ANA, XA	Serum, artificial and human saliva	This study

AuE—gold electrodes; SAM—self-assembled monolayer based on 11-mercaptoundecanoic acid, 11-mercapto-1-undecanol; BSA-pseudo-KA—pseudo-KA labeled with bovine serum albumin; KA-Ab—anti kynurenic acid antibody; Sec-Ab—anti-IgG-HRP antibody (HRP—horseradish peroxidase); FL-NS Tb^3+^/ZnO/CPE—carbon paste electrode modified with the flower-like nanostructures of zinc oxide doped with terbium; MIL-101 (Cr)/CPE—carbon paste electrode modified with chromium-based metal–organic frameworks; KYN—kynurenine; XA—xanthurenic acid; DP—dopamine, GL—glutamine; GLU—glutamic acid; ASA—ascorbic acid; SWV—square wave voltammetry; 3-HK—3-hydroxy-L-kynurenine; ANA—anthranilic acid; 3-HAA—3-hydroxyanthranilic acid.

**Table 3:** The recovery values obtained from DPV tests performed on real samples spiked with different concentrations of KA.

Biological Samples		KA (μM)		Recovery (%)	RSD (%) (n = 3)
		Added	Found		
Serum		25	24.63	98.53	4.53
		50	53.65	107.30	5.68
		100	102.47	102.47	1.44
Artificial saliva		50	57.41	114.82	3.54
		100	115.79	115.79	6.84
Human saliva	Raw	75	85.09	113.35	4.21
		100	88.68	88.68	8.22
	Diluted 1:1	100	93.11	93.11	2.86

**Table 4:** Analysis of KA in raw saliva samples collected from 15 subjects: 10 patients with the long COVID syndrome and five controls, using the optimized electrochemical sensor and the HPLC-UV as the control method.

Subject	HPLC Control Method			Electrochemical Sensor		
	KA Concentration (nM)	SD (nM)	RSD (%)	KA Concentration (nM)	SD (nM)	RSD (%)
Patient_1	41.73	0.84	2.00	42.87	0.28	0.66
Patient_2	35.98	0.71	1.96	36.85	1.75	4.76
Patient_3	46.18	1.11	2.39	43.36	0.36	0.83
Patient_4	49.76	0.57	1.15	53.23	0.37	0.70
Patient_5	50.42	0.67	1.32	54.04	0.16	0.29
Patient_6	25.79	0.29	1.11	27.56	0.51	1.85
Patient_7	28.97	0.76	2.62	29.62	0.51	1.71
Patient_8	67.58	0.85	1.26	62.69	0.93	1.48
Patient_9	75.72	1.40	1.84	77.43	1.39	1.80
Patient_10	46.50	1.61	3.46	47.37	1.56	3.29
Control_1	15.26	0.52	3.41	14.22	0.33	2.32
Control_2	7.59	0.30	3.99	7.16	0.15	2.16
Control_3	5.99	0.19	3.24	5.19	0.21	4.01
Control_4	8.71	0.21	2.47	8.22	0.32	3.89
Control_5	1.28	0.17	13.35	1.44	0.12	8.38

**Table 5:** The parameters generated by the ANOVA statistical analysis performed on the DPV data derived from KA levels determined in the raw saliva of 10 patients and five controls.

Source of Variation	SS	Df	MS	F	*p*-Value	F Crit
Between groups	0.483	1	0.483	0.00091	0.976	4.196
Within groups	14,950.1	28	533.9			
					p_theoretical_	
Total	14,950.6	29			0.05	

## Data Availability

The experimental data presented in this manuscript are available upon request and any further request should be addressed to the corresponding authors.
